# Optimizing Subtalar Arthrodesis: A Human Cadaveric Evaluation of a Novel Partially-Threaded Screw Combination in the Delta Configuration

**DOI:** 10.3390/medicina60060844

**Published:** 2024-05-22

**Authors:** Georgi Raykov, Stoyan Ivanov, Boyko Gueorguiev, Tatjana Pastor, Till Berk, Torsten Pastor, Ivan Zderic

**Affiliations:** 1 AO Research Institute Davos, 7270 Davos, Switzerland; st.ivanov@mu-varna.bg (S.I.); boyko.gueorguiev@aofoundation.org (B.G.); tatjana.pastor@spital.so.ch (T.P.); torsten.pastor@luks.ch (T.P.); ivan.zderic@aofoundation.org (I.Z.); 2 Department of Surgery, Cantonal Hospital of Uri, 6460 Altdorf, Switzerland; 3 Department of Orthopedics and Traumatology, Saint Marina Regional Hospital, Medical University Varna, 9002 Varna, Bulgaria; 4 Department of Plastic and Hand Surgery, Inselspital University Hospital Bern, University of Bern, 3007 Bern, Switzerland; 5 Department of Orthopedics, Traumatology and Reconstructive Surgery, University Hospital Aachen, 52074 Aachen, Germany; 6 Department of Orthopedics and Traumatology, Luzern Regional Hospital, 6110 Wolhusen, Switzerland

**Keywords:** subtalar arthrodesis, delta configuration, partially-threaded cannulated compression screws

## Abstract

*Background and Objectives*: Despite the established role of subtalar joint arthrodesis (SJA) for treatment of subtalar osteoarthritis, achieving bone union remains challenging, with up to 46% non-union rates. Adequate compression and stable fixation are crucial for successful outcomes, with internal screw fixation being the gold standard for SJA. The delta configuration, featuring highly divergent screws, offers stability, however, it can result in hardware irritation in 20–30% of patients. Solutions to solve this complication include cannulated compression screw (CCS) countersinking or cannulated compression headless screw (CCHS) application. The aim of this biomechanical study was to investigate the stability of a delta configuration for SJA utilizing either a combination of a posterior CCHS and an anterior CCS or a standard two-CCS combination. *Materials and Methods*: Twelve paired human cadaveric lower legs were assigned pairwise to two groups for SJA using either two CCSs (Group 1) or one posterior CCHS and one anterior CCS (Group 2). All specimens were tested under progressively increasing cyclic loading to failure, with monitoring of the talocalcaneal movements via motion tracking. *Results*: Initial stiffness did not differ significantly between the groups, *p* = 0.949. Talocalcaneal movements in terms of varus–valgus deformation and internal–external rotation were significantly bigger in Group 1 versus Group 2, *p* ≤ 0.026. Number of cycles until reaching 5° varus–valgus deformation was significantly higher in Group 2 versus Group 1, *p* = 0.029. *Conclusions*: A delta-configuration SJA utilizing a posterior CCHS and an anterior CCS is biomechanically superior versus a standard configuration with two CCSs. Clinically, the use of a posterior CCHS could prevent protrusion of the hardware in the heel, while an anterior CCS could facilitate less surgical time and thus less complication rates.

## 1. Introduction

Subtalar joint arthrodesis (SJA) is a well-established surgical procedure for treatment of hindfoot pathologies. Subtalar osteoarthritis—primary or secondary—represents its main indication [1,2,3,4]. Despite being recognized as a simple procedure, previous work reports up to 46% non-union rates [5,6,7,8,9]. Factors contributing to lower union rates include a history of smoking, alcoholism, avascular necrosis at the subtalar joint, prior failed arthrodesis, or a body mass index exceeding 30 kg/m^2^ [10,11,12]. Adequate compression and a stable fixation—especially in high-risk patients—are crucial to achieve union and improve surgical outcomes [13,14].

**Figure 1:**
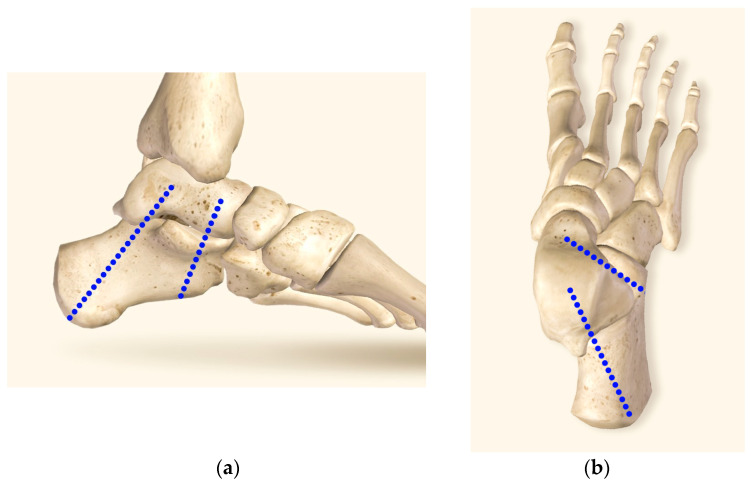
Visualization of the screw positioning for delta-configuration subtalar joint arthrodesis by means of two blue dot lines in medial (**a**) and dorsal (**b**) views. The insertion point of the posterior screw is located at the calcaneal tuberosity center. The screw trajectory is directed across the posterior facet of the subtalar joint at 90° angle, with the screw tip located in the talar dome center. The insertion point of the anterior screw is located at the lateral plantar aspect of the anterior calcaneus, 10 mm proximal to the calcaneocuboid joint. The screw trajectory is directed at 45° angle, passing dorsally and medially (parallel to the Chopart’s joint line) to the head and neck of the talus.

Screw fixation has been established as the gold standard for SJA [10]. Among two-screw configurations, the delta, or highly divergent configuration featuring a posterior and an anterior plantar screw (Figure 1), has been established as most stable [15,16]. Partially-threaded screws are favored for their ability to achieve compression between the bones [17]. However, a disadvantage of the delta configuration is that 20% to 30% of patients reportedly experience hardware irritation due to prominent screw heads [10,18,19]. One solution is to achieve sufficient countersinking of the cannulated compression screws (CCSs) to prevent head prominence and irritation [20]. Another solution is the use of cannulated compression headless screws (CCHSs) [19,21], delivering similar joint compression and pull-out force compared to CCSs [22,23]. On the other hand, CCHSs entail higher financial costs, are not readily available in all clinical centers, and their removal in non-union revision cases can be challenging, potentially prolonging surgical time and elevating complication rates [24].

**Figure 2:**
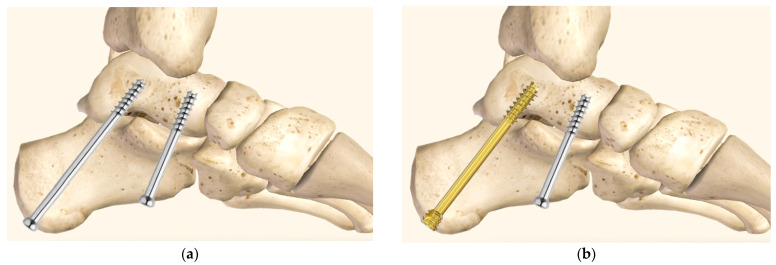
Visualization of delta-configuration subtalar joint arthrodesis in medial view, utilizing either two 6.5 mm partially-threaded cannulated compression screws (**a**) or a combination of one posterior partially-threaded 6.5 mm cannulated compression headless screw and one anterior partially-threaded 6.5 mm cannulated compression screw (**b**).

Therefore, the aim of this biomechanical study was to investigate the stability of a delta configuration for SJA utilizing either a combination of a posterior CCHS and an anterior CCS or a standard two-CCS combination (Figure 2), as to our knowledge, this has not been done in the literature so far.

Keeping in mind the advantages of each one of these screw types, it was hypothesized that a delta configuration for SJA utilizing a combination of a posterior CCHS and an anterior CCS would provide adequate stability, alleviate complaints of protruding hardware, decrease surgical time and thus complication rates, while reducing costs.

## 2. Materials and Methods

### 2.1. Specimens and Preparation

Twelve paired fresh-frozen (−20 °C) human cadaveric lower legs from one female and five male donors aged 69 ± 14 years (mean value ± standard deviation, range 60–89 years) were used. All donors gave their informed consent inherent within the donation of the anatomical gift statement during their lifetime. Any pathology or pre-existing trauma in the region of the ankle and hindfoot were ruled out via computed tomography (CT) scanning (Revolution EVO, GE Healthcare, Chicago, IL, USA) at a slice thickness of 0.63 mm.

A cut through the middle third of the calf, eight centimeters above the ankle joint, was performed, preserving the distal part of the syndesmosis at the ankle joint and enhancing physiological loading at the mortise [25]. All specimens were thawed at room temperature for 24 h prior to preparation and biomechanical testing.

**Figure 3:**
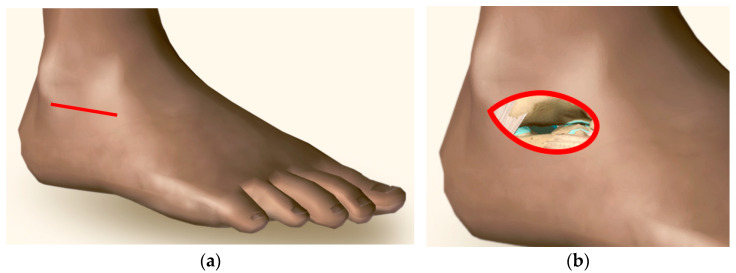
Visualizations of a specimen with sinus tarsi approach (**a**) and subtalar cartilage (marked in blue) to be removed through the sinus tarsi approach (**b**).

Subtalar joint preparation of the specimens was performed through a sinus tarsi approach, using an incision from the distal fibula over the anterior process of the calcaneus towards the base of the fourth tarsometatarsal joint (Figure 3) [26]. Special care was taken to prepare the joint surfaces, as described by Patel et al. [27]. The hindfoot was positioned and held in 5° valgus and dorsally flexed during screw insertion to provide adequate joint compression and to reduce the gap usually seen at the arthrodesis site on intraoperative radiographs [14,28].

**Figure 4:**
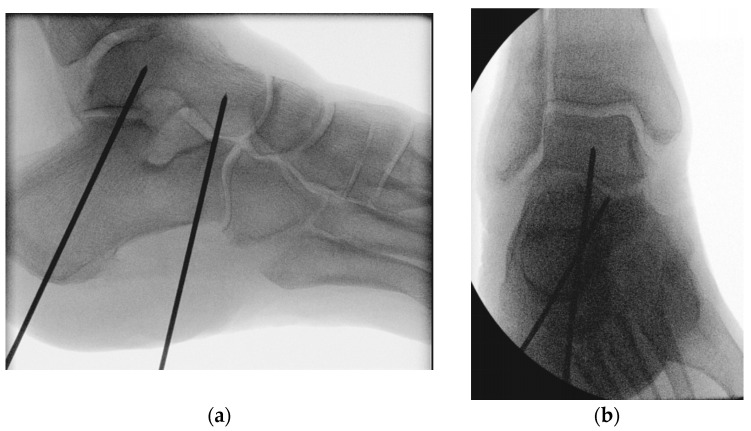
Radiographs visualizing the positioning of the two 2 mm guide wires used for screw insertion with delta configuration during subtalar joint arthrodesis in lateral (**a**) and anteroposterior (**b**) views.

Two 2 mm guide wires were placed and consequently joint fixation was conducted in a highly divergent delta configuration [29]. The insertion point of the first guide wire was located at the center of the calcaneal tuberosity. The wire was directed across the posterior facet of the subtalar joint at a 90° angle, with its tip located in the center of the talar dome [30]. The insertion point of the second guide wire was located at the lateral plantar aspect of the anterior calcaneus, 10 mm proximal to the calcaneocuboid joint. The wire was directed at an approximate angle of 45°, passing dorsally and medially (parallel to the Chopart’s joint line) to the head and neck of the talus [15]. Predrilling was then performed along the guide wires with a 4 mm gauge [31] (Figure 4).

**Figure 5:**
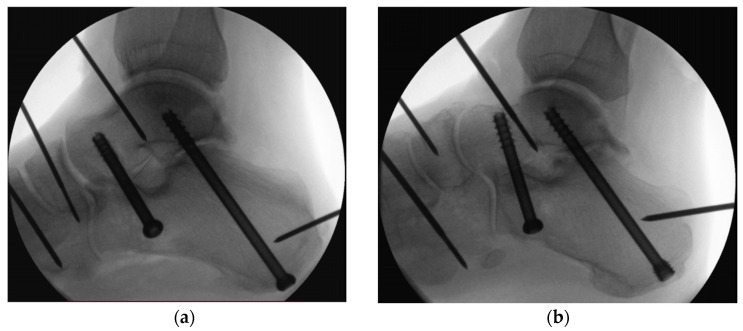
Radiographs visualizing the screw positions for delta-configuration subtalar joint arthrodesis using either two cannulated compression screws in Group 1 (**a**) or one posterior cannulated compression headless screw and one anterior cannulated compression screw in Group 2 (**b**).

The specimens were assigned pairwise to two groups for SJA using either two 6.5 mm partially-threaded CCS (Group 1) of 16 mm thread length (DePuy Synthes, Zuchwil, Switzerland) or one posterior partially-threaded 6.5 mm CCHS and one anterior partially-threaded 6.5 mm CCS (Group 2), both of 16 mm thread length (DePuy Synthes, Zuchwil, Switzerland) (Figure 5).

All screws were tightened manually applying the standard technique until a firm three-finger grip was felt [15,30].

In Group 1, the posterior CCS was countersunk to an appropriate depth, determined by the sloping tip of the countersinking instrument reaching the level of the surrounding bone [19].

The proximal 5 cm of the tibia and fibula of each specimen were embedded in polymethylmethacrylate (PMMA, SCS Beracryl D-28, Suter Kunststoffe AG, Fraubrunnen, Switzerland) after removal of the respective soft tissues. Finally, retroreflective marker sets were attached to the tibia, talus and calcaneus for motion tracking.

### 2.2. Biomechanical Testing

**Figure 6:**
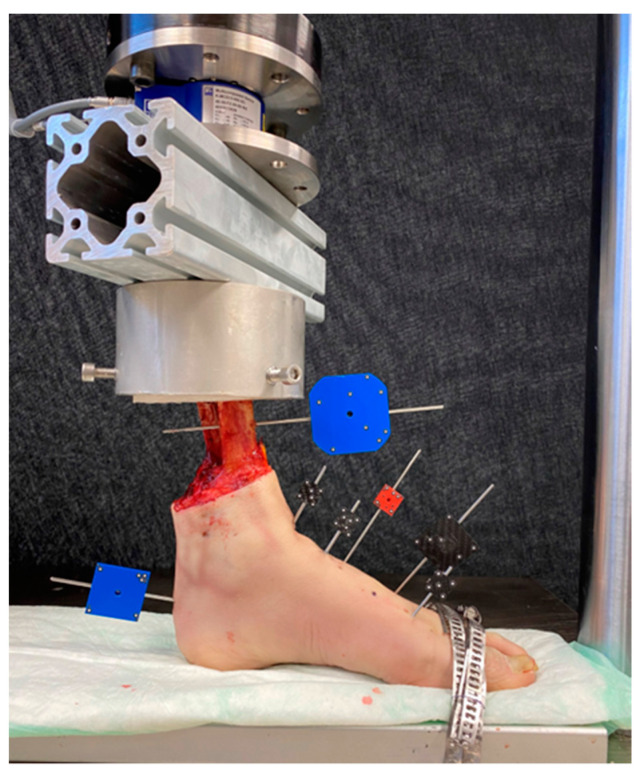
Setup with a specimen equipped with markers for motion tracking and mounted for biomechanical testing.

Biomechanical testing was performed on a servo-hydraulic testing machine (Bionix 858.20, MTS Systems, Eden Prairie, MN, USA) equipped with a 25 kN load cell. The setup with a specimen mounted for testing is presented in Figure 6. The tibial shaft was aligned to the machine axis to simulate a mid-stance foot position. The embedded proximal tibia and fibula were constrained to the machine transducer, while the foot was set on a flat surface, simulating a barefoot step. The forefoot was held with wires at the level of the proximal phalanges to provide specimen’s stability during testing under compression loading along the machine axis, allowing for unconstrained foot movements at the same time. The loading protocol commenced with a non-destructive quasi-static ramp from 50 N preload to 200 N at a rate of 15 N/s. Afterwards, progressively increasing cyclic loading to failure with physiologic profile of each cycle [32] was performed at 2 Hz. While the valley load of each cycle was maintained at a constant level of 50 N during testing, the peak load, starting at 200 N (cycle 1), was monotonically increased at a rate of 0.1 N/cycle. Previous studies established the viability of cyclic testing with monotonically increasing load levels [33,34]. The test was interrupted as soon as the machine actuator reached 5 mm displacement, considered as an appropriately defined test stop criterion resulting in considerable catastrophic damage of the bone-implant constructs for sound retrospective data analysis. The protocol for dynamic loading implemented the worst-case scenario of a clinical situation without subtalar bone fusion of an 80 kg patient starting with 25% partial weight bearing (WB) 6 weeks postoperatively and increasing it to full WB within the next 6 weeks, i.e., until the 12th week post-surgery, assuming an activity of 1000 loading events per week. The further increase of the dynamic loading during biomechanical testing was performed because of the aimed catastrophic specimen’s failure.

### 2.3. Data Acquisition and Evaluation

Machine data in terms of axial displacement and axial force were recorded at 128 Hz. Based on the initial quasi-static ramp, initial stiffness was evaluated from the linear slope of the ascending force–displacement curve within a range of 20–200 N.

The co-ordinates of the attached retroreflective markers were continually captured by a stereographic camera system (Aramis SRX; Carl Zeiss GOM Metrology GmbH, Braunschweig, Germany) for motion tracking to investigate the relative movements of the talus and calcaneus in all six degrees of freedom. Based on the motion tracking data, the angular talus–calcaneus movements were evaluated in terms of varus–valgus, flexion–extension, and internal–external rotation. In addition, the magnitude of the translational movement at the most inferior aspect of the subtalar joint—defined as displacement—was calculated. The values of these outcomes were analyzed after 2000, 4000, 6000, 8000 and 10,000 cycles in peak loading condition. The last number of cycles was specified as the biggest rounded number when none of the specimens had yet failed. Furthermore, 5° varus–valgus, flexion–extension, and internal–external rotation, were defined as criteria for clinically relevant construct failures, and the corresponding numbers of cycles and peak load until fulfillment of each one of these three criteria—defined as cycles to failure and failure load, respectively—were evaluated. In addition, cycles to failure and failure load were assessed according to a criterion for earliest failure, representing a combination of the three criteria for clinically relevant failure.

Mediolateral X-rays were taken at the start of every cyclic test and then every 250 cycles under peak loading using a triggered C-arm (Siemens Arcadis Varic, Siemens AG, Erlangen, Germany). The collected images were used to track the progress until failure of each specimen. The specimens in both groups were inspected after testing radiologically and visually after dissection.

Statistical analysis was carried out using the SPSS software package (V. 27, IBM, Armonk, NY, USA). Evaluation and verification of the normal data distribution was conducted by utilizing the Shapiro–Wilk test. To identify significant distinctions within each study group and between the groups, the Paired-Samples *t*-test, Wilcoxon Signed-Rank test for longitudinal pooled data, and General Linear Model Repeated Measures test were employed. Level of significance was set to 0.05 for all statistical tests.

## 3. Results

### 3.1. Initial Stiffness

No significant difference was detected for initial stiffness between Group 1 (172.3 ± 41.2 N/mm) and Group 2 (173.7 ± 59.7 N/mm), *p* = 0.949.

### 3.2. Talus–Calcaneus Movements

The outcomes related to talus–calcaneus movements evaluated over the five time points after 2000, 4000, 6000, 8000 and 10,000 cycles are presented in Table 1. The values of each separate outcome increased significantly over the five time points, *p* ≤ 0.007. Varus–valgus, internal–external rotation, and displacement were significantly bigger in Group 1 versus Group 2, *p* ≤ 0.026. Flexion–extension was not significantly different between the groups, *p* = 0.078.

### 3.3. Cycles to Failure

The numbers of cycles to failure and the corresponding failure loads until reaching the predefined clinically relevant failure criteria are summarized in Table 2. Although there were no significant differences between the groups for cycles to failure and failure load according to the 5° flexion–extension and internal–external rotation criteria, *p* = 0.179, the specimens in Group 2 demonstrated significantly higher number of cycles to failure and failure load until reaching 5° varus–valgus, *p* = 0.029. There was a strong trend towards higher number of cycles to failure and failure load with regard to the criterion for earliest failure in Group 2 versus Group 1, *p* = 0.051.

### 3.4. Modes of Failure

**Figure 7:**
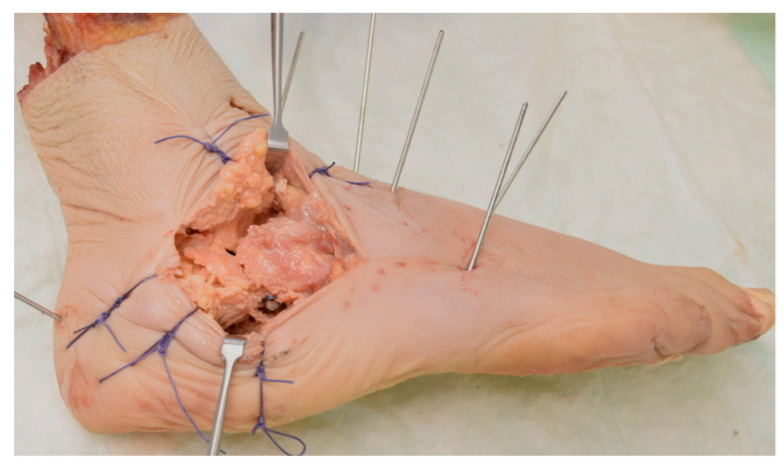
Depression-type intra-articular calcaneal fracture with loosening and fracture along the anterior screw observed post testing after catastrophic failure of a specimen in Group 2.

**Figure 8:**
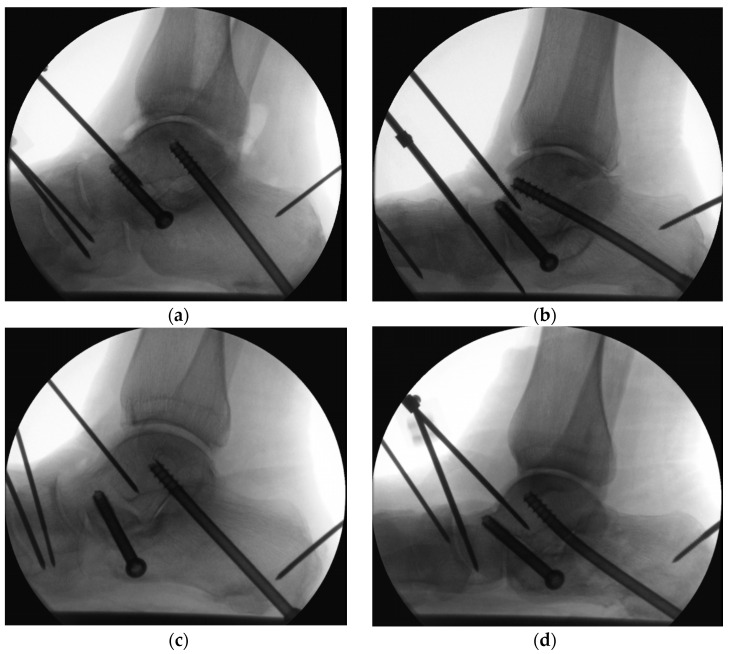
Radiographs visualizing catastrophic failures: (**a**) loosening of the anterior and posterior CCSs plus depression-type intra-articular calcaneal fracture in Group 1; (**b**) loosening of the 2 screws with bending of the posterior CCHS plus posterior calcaneal tuberosity fracture in Group 2; (**c**) loosening of the anterior screw plus fractures of the anterior process and posterior tuberosity in Group 1; (**d**) loosening of the anterior screw and bending of the posterior CCHS plus posterior calcaneal tuberosity fracture in Group 2.

Progressive loosening of the screws was observed radiologically in all specimens during cyclic testing. Loosening with pull-out of the anterior and posterior screws were also observed post testing (Figure 7). Bending of the posterior screw was indicated in both groups, however, with a higher frequency in Group 2: there were 4 bent CCHSs in Group 2 versus 2 bent CCSs in Group 1 (Figure 8).

## 4. Discussion

The current study investigated the stability of a delta configuration for SJA utilizing either a novel combination of a posterior CCHS and an anterior CCS or a standard two-CCS configuration. In terms of displacement, varus–valgus and internal–external rotation movements, as well as cycles to failure and failure load, the novel screw combination was related to significantly higher stability. This finding demonstrates that the posterior CCHS contributes more than the posterior CCS to the SJA construct stability—keeping in mind that the anterior screw fixation was identical in both groups. Both groups, however, revealed similar initial stiffness of the delta configuration.

A greater proportion of the CCHSs demonstrated superior resistance to higher loads prior to failure when compared to the corresponding CCSs. This suggests that the CCHS configuration, with its dual fixation aspects in the talus and calcaneus, likely contributed to the superior performance of Group 2 versus Group 1.

The overall enhanced stability observed in the delta screw configuration is likely attributed to its broader coverage area, which helps prevent relative movement between the talus and calcaneus due to the greater distance between the screws. Another important advantage of this configuration is the preservation of the posterior facet bone surface that is able to promote successful subtalar fusion, as previously reported [29]. In a human cadaveric study exploring the impact of screw placement on the joint rotation and fusion site stiffness, Hungerer et al. concluded that the delta configuration significantly reduced both frontal- and transverse-plane joint rotations [16]. Furthermore, it notably enhanced the stiffness of the fusion site when compared to parallel screw configurations. The high initial stiffness in both groups of the current study is in agreement with these findings.

Some authors are in favor of using partially-threaded over fully-threaded screws because of their higher initial compression rate. While in their study Boffeli and Reinking implemented a delta configuration as a combination of a posterior partially-threaded and an anterior fully-threaded screw, they hypothesized the advantage of using an anterior partially-threaded screw that could provide better compression and improve the union rate at the anterior facet [29]. Taking this into account, we opted for using an anterior partially-threaded screw in the current study.

Compared to CCSs, CCHSs are technically more demanding, especially in case of hardware removal [24]. Previous work reported that using CCHSs in calcaneal osteotomies reduced the revision rate by 40% [19]. In contrast, Sayres highlighted the superiority of using two cannulated headed screws in calcaneal osteotomies when compared to other fixations, stating that it is technically less demanding, provides adequate compression, and has a relatively low cost [24]. On the other hand, the CCHS cost is nearly two-fold than that of CCS and the former is not available in all hospitals. That is why we opted for a posterior CCHS—in order to reduce hardware irritation in the heel—and an anterior CCS to reduce costs and technical difficulty, while providing similar stability as a CCHS.

All used screws in the present study were with a 6.5 mm diameter, as they feature a comparable pull-out force as 7.3 mm or 8.0 mm cannulated screws [16,35,36].

Based on the study by Assari et al.—concluding that (1) screw over-tightening does not correlate with the increase of compression force and (2) demonstrating that the tightening torque applied by the surgeon can be a misleading measure of the compression force [21]—care was taken in the current study to avoid any over-tightening of the screws during insertion, especially of the anterior one, considering the highly cancellous nature of the bone within the anterior talus and calcaneus.

Risk factors such as smoking, alcoholism, diabetes, and age have been proven to increase the risk of subtalar non-union. In such patient clusters it is advisable to attempt achieving a SJA which is initially as stable as possible. Riedl et al. stated that improved fixation could be achieved by optimization of screw placement patterns and use of three screws instead of two [13,23]. Although biomechanical studies support the superiority of a three-screw construct over a two-screw one regarding stability and compression, Wirth et al. pointed out that the two-screw fixation is clinically preferable due to a shorter operation time, lower risk of wound complications, and eventual need for implant removal [11]. A three-screw fixation is advisable in revision SJA cases.

The clinical protocol for SJA was implemented in the current study and the subtalar cartilage was removed in all specimens through the sinus tarsi approach. Bone-to-bone contact between the talus and calcaneus was achieved, providing better stability of the screw fixation [26,27,36].

This study has some limitations similar to those inherent to all human cadaveric investigations, incapable to completely simulate an in vivo environment. A limited number of specimens were tested, resulting in restriction of the translation to generalized clinical applications. As a result, some differences in the biomechanical performance between the groups could be substantiated only as a statistical trend. Next, the present study represents biomechanical conditions without consideration of bone healing and bone growth around the screws and in the subtalar joint, that would have enhanced the talus–calcaneus fixation over time. Further, the reproduction of SJA was not identical to an in vivo one, as there was no actual bone fusion. On the other hand, the particular sequence of cartilage removal and screw fixations ensured consistent reproducibility of the arthrodesis pattern. Moreover, the biomechanical simulation utilized in this study mostly involved axial loading of the foot. This simplified loading scenario did not fully recreate the complex forces experienced during the human gait. However, the simulation allowed for an unconstrained movement of the hind- and midfoot, while fixating only the forefoot with wires to the testing table. Furthermore, according to the SJA protocol of Ferrao, postoperative immobilization should last 6 weeks with a non-WB cast and then for 6 weeks with an immobilizer boot, while partial WB is allowed starting from the 6th week onwards and later progressively increasing the load from the 8th until the 12th week postoperatively [26]. Hence, during immobilization, the foot would primarily be axially loaded. Adequate osseus fusion can take 12 to 16 weeks of time, which is why our results are relevant, especially in a worst-case scenario of a clinical situation without bone fusion.

## 5. Conclusions

A delta-configuration SJA utilizing a posterior CCHS and an anterior CCS is biomechanically superior versus a standard configuration with two CCSs and implements the benefits of the latter enhancing a successful subtalar joint fusion. Clinically, the use of a posterior CCHS could prevent protrusion of the hardware in the heel, while an anterior CCS could facilitate less surgical time and thus less complication rates, as well as reduced costs. Further clinical studies are required to confirm the results of this biomechanical study.

## Figures and Tables

**Table 1:** Outcomes related to talus–calcaneus movements, evaluated over the five time points after 2000, 4000, 6000, 8000 and 10,000 cycles in each separate group and presented in terms of mean value and standard deviation, together with *p*-values from the statistical comparison between the groups.

Outcome	Group	Cycles					*p*-Value
		2000	4000	6000	8000	10,000	
Varus–valgus [°]	1	1.37 (0.75)	2.89 (1.47)	4.38 (2.31)	6.34 (3.85)	8.27 (5.00)	<0.001
	2	0.96 (0.60)	1.72 (1.14)	2.48 (1.28)	3.52 (1.91)	5.29 (4.38)	
Flexion–extension [°]	1	0.65 (0.72)	1.38 (0.80)	2.45 (1.39)	3.18 (1.52)	4.82 (2.66)	0.078
	2	0.56 (0.64)	1.00 (0.93)	1.67 (1.28)	2.77 (1.94)	4.17 (3.07)	
Internal–external rotation [°]	1	0.51 (0.38)	1.16 (1.10)	1.87 (1.73)	1.74 (1.30)	2.46 (1.26)	0.026
	2	0.45 (0.70)	0.69 (1.01)	0.95 (1.21)	1.42 (1.44)	3.07 (2.68)	
Displacement [mm]	1	1.51 (0.94)	3.02 (1.57)	4.89 (2.72)	6.89 (4.84)	9.31 (5.36)	<0.001
	2	0.86 (0.86)	1.49 (1.26)	2.30 (1.50)	3.61 (2.22)	6.81 (5.82)	

**Table 2:** Cycles to failure and failure load in each separate group, presented in terms of mean value and standard deviation, together with *p*-values from the statistical comparison between the groups.

Criterion	Group	Cycles to Failure	Failure Load	*p*-Value
5° Varus–valgus	1	9169 ± 5880	1117 ± 788	0.029
	2	14,053 ± 6225	1605 ± 823	
5° Flexion–extension	1	10,368 ± 2643	1237 ± 464	0.179
	2	13,539 ± 5696	1554 ± 770	
5° Internal–external	1	14,348 ± 3746	1635 ± 575	0.840
	2	14,766 ± 4880	1678 ± 688	
Earliest failure	1	8068 ± 3781	1007 ± 578	0.051
	2	10,439 ± 2523	1244 ± 452	

## Data Availability

All data relevant to the study are included in the article.
